# No longer *‘flying blind’*: how access has changed emergency mental health care in rural and remote emergency departments, a qualitative study

**DOI:** 10.1186/s12913-015-0839-7

**Published:** 2015-04-14

**Authors:** Emily Saurman, Sue E Kirby, David Lyle

**Affiliations:** Broken Hill University Department of Rural Health, University of Sydney, PO BOX 457, Broken Hill, NSW 2880 Australia

**Keywords:** Access, Mental health, Emergency care, Telehealth, Rural and Remote, Australia, Qualitative study

## Abstract

**Background:**

Mental health presentations are considered to be a difficult aspect of emergency care. Although emergency department (ED) staff is qualified to provide emergency mental health care, for some, such presentations pose a challenge to their training, confidence, and time. Providing access to relevant and responsive specialist mental health care can influence care and management for these patients. The Mental Health Emergency Care-Rural Access Program (MHEC-RAP) is a telepsychiatry program that was established to improve access to specialist emergency mental health care across rural and remote western NSW, Australia.

**Method:**

This study uses interviews with ED providers to understand their experience of managing emergency mental health patients and their use of MHEC-RAP. The lens of access was applied to assess program impact and inform continuing program development.

**Results:**

With MHEC-RAP, these ED providers are no longer ‘flying blind’. They are also more confident to manage and care for emergency mental health patients locally. For these providers, access to specialists who are able to conduct assessments and provide relevant and responsive advice for emergency mental health presentations was valued. Assessing the fit between the consumer and service as a requirement for the development, evaluation, and ongoing management of the service should result in decisions about design and delivery that achieve improved access to care and meet the needs of their consumers. The experience of these providers prior to MHEC-RAP is consistent with that reported in other rural and remote populations suggesting that MHEC-RAP could address limitations in access to specialist care and change the provision of emergency mental health care elsewhere.

**Conclusion:**

MHEC-RAP has not only provided access to specialist mental health care for local ED providers, but it has changed their practice and perspective. MHEC-RAP could be adapted for implementation elsewhere. Provider experience confirms that the program is accessible and offers insights to those considering how to establish an emergency telepyschiatry service in other settings.

## Background

There is a consistently reported gap between the need for mental health services and actual mental health service use, not only in Australia but overseas [1-5]. Although there is little difference in the prevalence of mental health problems across urban and rural populations in Australia, rural and remote communities have poorer access to and lower use of specialist mental health services [6,7]. This makes the provision of emergency mental health care problematic and is associated with poorer mental health outcomes, such as higher Kessler Psychological Distress Scale scores and higher suicide rates [8-10].

Emergency Departments (EDs) and General Practitioners (GPs) are common providers of mental health care in rural communities [11-16]. Although GPs and ED staff are qualified to provide this care, responding to emergency mental health presentations (patients with acute acerbations of their condition, in crisis, or endangering themselves, others, or their reputation) pose diagnostic and management challenges for local providers who may lack specific mental health training, confidence, or time for emergency mental health presentations. In some communities, the facility may not be adequately equipped for the safe assessment and monitoring of these patients whose needs differ from those with physical ailments [15,17-22].

In Australia, the National Strategic Framework for Rural and Remote Health, along with the National Mental Health Plan and complementary State Action Plans all aim to improve access to care [23-27]. In line with national and state policies, the Mental Health Emergency Care-Rural Access Program (MHEC-RAP) was established in the Western NSW and Far West Local Health Districts (WNSW/FWLHD) of NSW to improve access to specialist emergency mental health care, safety and service coordination, and patient outcomes [27-29].

MHEC-RAP has been operating since 2008. It was not the first, nor the only, telepsychiatry service to be developed and used in Australia [30,31]. But it was the first to provide 24-hour access to a dedicated, regionally-based team of mental health specialists offering timely information and support, emergency telephone triage, and video assessment for all providers, patients, and residents needing urgent mental health care in rural and remote communities. The MHEC-RAP evaluation has already reported findings from earlier studies that demonstrate use of the program by those in need and examined clinical service activity from the area EDs [32,33]. This was the first evaluation to use a time and motion study method to assess program efficiency and to present a translatable program model for transferability [34,35].

One aim of MHEC-RAP is to improve access. Providing access to relevant and responsive specialist mental health care can have significant implications for the patient, their family, local providers, emergency services, and the local health service. The aim of this study was to examine the impact of MHEC-RAP on providing access to specialist care through the experience of local providers and was guided by six concepts of access (accessibility, availability, acceptability, affordability, adequacy, and awareness) [36].

## Methods

This part of the MHEC-RAP evaluation was approved by the Greater Western Human Research Ethics Committee and adheres to the National Statement on the Conduct of Human Research by the Australian National Health and Medical Research Council, Project Number: HREC/13/GWAHS/9.

### Study design

This study applied a qualitative method to interpret the experience of the local ED providers and examine the impact of MHEC-RAP on the provision of access to specialist emergency mental health care. The analysis was guided by six concepts of access [36]. Their experiences can be used to inform further program development, implementation, and transferability.

Semi-structured interviews were conducted with providers of emergency mental health care from communities across the WNSW/FWLHD. Interviews would be conducted with consenting participants until there was a saturation of findings. Saturation was determined to be achieved when no new or differing information was revealed across the interviews. Participants were asked about providing emergency mental health care, the resources available to them, the positive experiences, as well as the challenges and other considerations of providing this care in rural and remote western NSW.

The questions were developed in consideration of the theory of access but allowed for open response from each provider to share their experience and perception of emergency mental health care and MHEC-RAP [36]. Initially, broad questions were used to explore their experience which later transitioned to focus on MHEC-RAP after the program was mentioned in the interview. If it was not mentioned, the provider was directly asked about their knowledge and experience of the program. This question design, and the bracketing of previous knowledge, enabled ES to conduct interviews that informed current understanding of experience and perception of emergency mental health care and the impact of MHEC-RAP on access.

### The program

**Figure 1:**
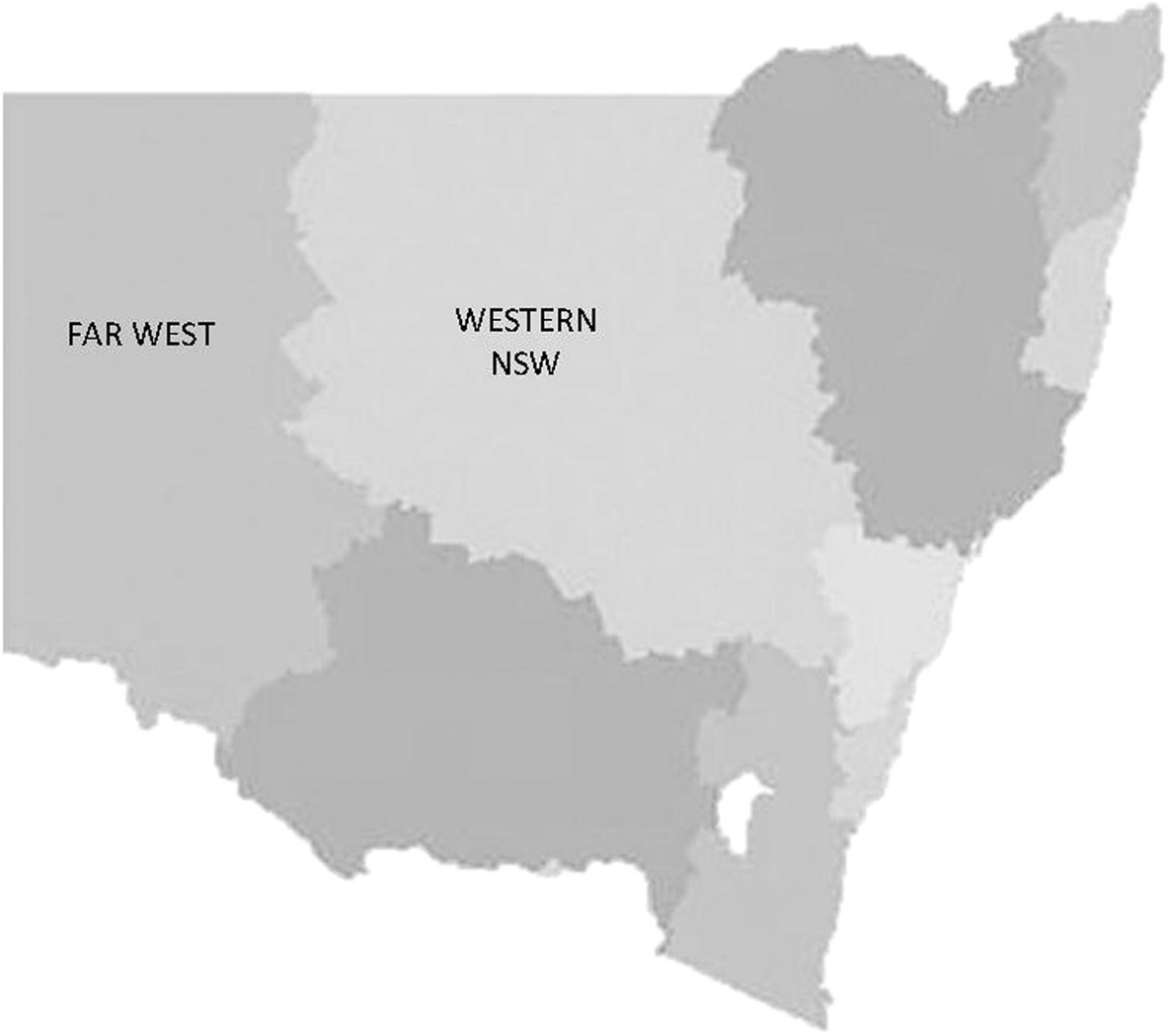
Map of NSW divided by Local Health District - FarWest LHD and Western NSW LHD identified.

MHEC-RAP was developed to improve access to mental health specialists for anyone needing emergency mental health care or assistance in the WNSW/FWLHD. Combined, these health districts serve approximately 300,000 residents living across 445,000 km^2^ of regional, remote, and very remote countryside [37]. Providing care from one program for this population is challenging because this area is similar in size to Germany or the state of Montana (Figure 1). MHEC-RAP employs telehealth technologies and a freecall number to provide timely information and clinical services from a dedicated team of mental health specialists. Further detail of the program model and structure is available in a previous publication [35].

### Recruitment

Rural and remote providers, specifically ED staff (managers and nurses) and GPs, were invited to participate. They were purposely chosen because the video assessment equipment used to connect with MHEC-RAP is located in the hospital EDs and for many rural or remote communities, the local GPs also work in the local hospital.

Based on the patterns of use of MHEC-RAP clinical services in 2011, 18 communities from the 48 with hospital facilities across the region were identified for recruitment [32]. Communities with high use received 50 or more MHEC-RAP clinical services that year, medium use was 10–49 services, and low use was below 10. Six communities were identified from each of the remoteness structure categories; remote and very remote communities were combined for this. All GPs from the identified communities were invited for participation along with four ED staff from each hospital via their hospital manager; 169 providers in total. The invitation included an information letter as well as consent and withdrawal forms and a pre-addressed and stamped return envelope. All participants were informed of the purpose of the interview in the invitation letter and this was again reviewed on the day of the interview. No incentives were provided to eliminate any possible coercion to participate. The recruitment strategy acknowledged that there could be a high drop-out rate because some invitations could be lost, misplaced, or ignored thereby justifying the large recruitment population and resulting in a convenience sample of the population. All those invited were free to withdraw their participation without prejudice prior to the interview.

Thirteen consenting responses were received from seven communities across the region. Respondents were male and female, of rural and urban background, and served as a provider for varying lengths of time. All 13 consenting respondents were approached for an interview (an 8% response rate); 12 were ED staff and one was a GP, no additional GPs responded. No identifiable information, such as community, was reported to maintain participant confidentiality; though the participants were free to disclose their participation to others.

### Data collection

Twelve of the consenting participants from six of the identified communities were interviewed; one respondent withdrew. ES conducted all 12 interviews in September and October 2013. All but two interviews were conducted face-to-face; telephone interviews were arranged for those who were unable to attend their face-to-face interview due to unplanned commitments on the day. The interviews were commonly conducted in the health service setting; one interview was in a private home and another in a public venue as requested by those participants. Each interview was digitally recorded for transcription and analysis. All interviews were private and the recordings were no longer than one hour. Notes were also recorded after each interview. All participants were available by telephone for subsequent contact to clarify any query to their response as needed.

### Analysis

Each interview was transcribed by ES within two days and initial analyses begun immediately; this helped to determine saturation of findings. Although the response rate was low and the researchers were permitted to extend the invitation for participation to other communities, this was not considered necessary with a saturation of findings. ES conducted all data analyses; the transcripts and resulting analyses were reviewed and discussed with SK and DL to confirm usefulness, rigour, and quality of findings. Coding and analysis were conducted using paper-based methods and NVivo [38]. For reporting purposes, locations were given a number associated with its remoteness category and the number of participant interviews conducted. Participant responses were identified by their community number and assigned a letter (A, B, C) which was determined by the order of the interviews as they were conducted. For instance, the third participant interviewed in community 3 would be identified as 3C (Table 1).

**Table 1:** **Characteristics of each community and those interviewed**

**Community**	**Interviews**		Remoteness category*	Level of use of MHEC-RAP**
	**N = 12**	**Provider**		**(High/Medium/Low)**
1	3	ED/GP	Inner Regional	High
2	1	ED	Inner Regional	High
3	3	ED	Outer Regional	Medium
4	1	ED	Outer Regional	High
5	3	ED	Remote	Low
6	1	ED	Remote	Medium

*as determined by the Australian Standard Geographical Classification.

**actual use of MHEC-RAP clinical services from the ED in 2011; High ≥ 50 clinical services, Medium = 10-49, Low < 10.

The analysis presented here examined the provider experience of MHEC-RAP. It was structured using a theoretically concept-driven content analysis that aligned to the six concepts of access. Access is about providing the right care, from the right provider, at the right time, and in the right place. Penchansky and Thomas define ‘access’ as the degree of fit between the consumer and the service [36]. Their theory of access incorporates and addresses five specific concepts of fit; accessibility, availability, acceptability, affordability, and adequacy (Table 2).

**Table 2:** **The concepts of access**

**Concept of access**	**Definition**	**Concept components and examples**
Accessibility*	Location	An accessible service is within reasonable proximity to the consumer in terms of time and distance.
Availability*	Supply and demand	An available service has sufficient services and resources to meet the volume and needs of the consumers and communities served.
Acceptability*	Consumer perception	An acceptable service responds to the attitude of the provider and the consumer regarding characteristics of the service and social or cultural concerns. For instance, a patient’s willingness to see a female doctor may determine whether a service is acceptable or not.
Affordability*	Financial and incidental costs	Affordable services examine the direct costs for both the service provider and the consumer.
Adequacy* (Accommodation)	Organisation	An adequate service is well organised to accept clients, and clients are able to use the services. Considerations of adequacy include hours of operation (afterhour services), referral or appointment systems, and facility structures (wheelchair access).
Awareness**	Communication and information	A service maintains awareness through effective communication and information strategies with relevant users (clinicians, patients, the broader community), including consideration of context and health literacy.

*The five concepts of access identified by Penchansky and Thomas [36]. Penchansky, R. and J. W. Thomas. “The Concept of Access: Definition and Relationship to Consumer Satisfaction.” *Medical Care* **19**(2): 127–140.

**Awareness, a sixth concept that may influence access.

The theory was modified with the inclusion of a sixth concept – awareness. Awareness was identified as an important concept of access from interviews during the initial study of MHEC-RAP. In these interviews providers and patients spoke about how such a program is needed and could be useful, but that they did not know anything about it. They did not know that the program existed, what it did, why and how they would use it, and how to share this information with others who would benefit from the program. Informal conversations with colleagues and other health providers confirmed the importance of awareness to making a health service useful and effective. Consumers could better use services if they were simply aware of them in the first place.

ES was part of the initial study of MHEC-RAP in 2008 [39]. To understand the impact of MHEC-RAP on access and the experience of local providers five years later, ES applied Husserl’s approach to bracket her “beliefs, preconceptions, and prejudices” about the program and its influence on access to be open to the current experience [40-43]. Bracketing is putting one’s preconceptions aside to limit the influence of foreknowledge on the data collection, analysis, and interpretation.

‘Awareness’ is the missing concept of access and is more than knowing that a service exists, it includes identifying that the service is needed, knowing who the service is for, what it does, when the service is available, where and how to access it, why the service would be used, how to use it, and maintaining that knowledge. Others have also considered ‘awareness’ when applying the theory of access [44]. Although each concept is an individual consideration of fit between the service and the consumer, they are inter-connected and naturally overlap. All six concepts should be considered when applying the theory of access to the development or evaluation of health services and other programs more generally. Analysis and the reporting of the results of this study were structured to align with all six concepts. A before and after category was also applied to help determine effect and impact of the program.

## Results

The general accounts of providing emergency mental health care in rural and remote EDs varied. Some providers had positive experiences - when patients received timely and appropriate care, but everyone had negative experiences - dealing with patients who were variously abusive, sedated and involuntarily admitted to hospital (scheduled), absconding, or threatening harm, with no one to help. Mental health presentations were considered to be a difficult aspect of emergency care challenging their training and causing stress and disruption.

*We’re just so focused on people that are bleeding and coughing and everything else … there’s just not a lot of training [for mental health]. -6A*

*If [mental health is] not managed well, [it] can be devastating for the whole department and other patients and a whole lot of other things ‘cause they do tend to disrupt [the whole department]. -5B*

Residing and working in a rural or remote community also influenced their experience of providing emergency mental health care. This was true for those who were born or raised locally and those who were new in town. Some reflected on knowing their regular patients and their different relationships within the broader community (nurse, friend, relative). Those who had worked in metropolitan hospitals reflected on the differing access to care and resources. Everyone spoke about the limited resources available to them for managing mental health emergencies. Limitations ranged from the layout of the ED to the availability of local mental health workers, other hospital staff, or even the police for assistance when needed. In the end, everyone wanted to do the best that they could for their patients.

*This being a small rural town, we know the majority of them and we can have a really good relationship with them and they feel secure by coming here and talk to us because they know who we are, they know our role, they know that we are here to help them, and the majority of them are very accepting of the service that we provide for them because we have a really good rapport with them. -3C*

*The design of our ED makes it even worse than it is, we’re just not designed for a lot of things, it’s not just mental health, but mental health’s so difficult. -2A*

*I called the local mental health care team who couldn’t do anything because it was Friday afternoon and none of them work on the weekend. -1B*

MHEC-RAP was introduced to provide access to specialist emergency mental health care. The local providers’ experience of MHEC-RAP has been interrogated using six concepts of access and identifies a change in practice.

### Accessibility

MHEC-RAP had changed accessibility because local providers were now able to access a mental health specialist for immediate assistance, by telephone or video link, no matter which community in western NSW they were from. They did not have to send the patient to another facility or wait for a specialist to arrive to get assistance or have a patient assessed.

*They’re there, there’s video conferencing, you can talk to them, they’re there 24-hours a day. -4A*

*It makes such a difference being able to have somebody on the other end of the phone. -5B*

*The nearest mental health is actually based in [a town over an hour’s drive away]. … When somebody comes in, they’re reviewed, they’re assessed, they’re immediately talking to MHEC-RAP. -6A*

### Availability

MHEC-RAP was valued as a resource with specialist knowledge available to respond to the needs of local providers and ease the demands placed upon them during emergency mental health presentations. For some of the staff, MHEC-RAP was not only about getting specialist help for their patients, but about responding to their personal need for support in clinical decision making. There were some incidents when a request for assistance from MHEC-RAP was met with delay, but it was acknowledged that MHEC-RAP usually responds in a timely fashion.

*For us, having someone to just talk to, having someone that you can say ‘look, I’ve got this situation, what do you suggest, how do you think I should handle it’. …I’m quite happy with what [MHEC-RAP are] doing, you know, they’re giving me what I need. –3B*

*We can still ring [MHEC-RAP], get advice over the phone, put the person over in front of the tv camera, they can still do a face-to-face interview. … because they’re busy, it’s difficult when they’re not available and so sometimes that’s a bit frustrating, that you’re trying to get on to them. But on the whole I really like them. I think they’re a really good service. So it saved our bacon a few times. -1A*

### Acceptability

Although *“it doesn’t solve everything” -5C*, MHEC-RAP was acceptable to these providers. It was helpful and supportive, a constant and easy resource. A few commented about being generally uncomfortable with mental health and that for them, MHEC-RAP provided trained specialists to help with these emergencies. MHEC-RAP also improved their confidence to provide mental health care locally, and the hours associated with an emergency presentation, both waiting for and managing care, were reduced. With MHEC-RAP, providers were able to get their patients seen in a timely manner thereby reducing the possible anxiety, aggression, or absconding as well as helping to provide responsive care. Providers felt supported in their decisions and management of patients, and patients were seen to receive appropriate care and to have better outcomes.

*If we can get them MHEC-RAPped and they have someone to talk to, then they’ll find they go home with a plan. …I love it because I really, I don’t like dealing with [mental health] and I’d rather them talking to someone that’s gonna help them. -3A*

*Before MHEC-RAP there was, patients used to sit in emergency departments for much much longer with no definitive care. -1A*

*[MHEC-RAP has] basically reduced the crisis. …They come in and instead of sort of waiting for our doctors to come and da-da-da, we can actually get the process happening straight away and everything just calms down. Everything’s just alleviated and we can get the right treatment for them rather than managing them and waiting for our doctors to turn up. -3B*

MHEC-RAP was also acceptable because ED providers could leave the patient with the MHEC-RAP specialist over the video link, if appropriate, allowing for privacy and permitting the local staff to continue providing care to others. A MHEC-RAP video assessment was considered for use for almost every mental health presentation except those particularly violent or under the influence of substances. Even patients who were having auditory hallucinations had no problem using MHEC-RAP. There were a couple accounts of patients who did not want to be assessed by MHEC-RAP because they were uncomfortable with the technology or had a preferred psychiatrist, but MHEC-RAP was still able to provide assistance to the local provider for those patients. For a few providers, MHEC-RAP became their standard practice for emergency mental health presentations; some EDs were even told by the local doctors to have a patient seen by MHEC-RAP before they became involved.

*I can leave them in the room and then they can either talk to [MHEC-RAP] over the phone or … have a chat to them via the tele[vision] and I just think that’s brilliant. -3B*

*Especially the drug-induced psychosis type people that come in and they just want to bash the wall or hit people or bite and hit and screaming, … they’re not in any shape to talk to anybody so we don’t usually use [MHEC-RAP] for that. -1C*

*Everyone that’s acutely unwell from the mental health perspective … are reviewed by MHEC-RAP. -6A*

Nonetheless, there were reservations expressed about the program. One provider felt that it should not become a replacement for local specialist care because there is a need for local face-to-face care. A note of caution was expressed if the MHEC-RAP clinicians appeared to not fully understand the local context or situation, and concern was raised when the advice given was dissonant to local clinical judgement. However, if local providers were concerned, they felt comfortable enough to talk with MHEC-RAP about it.

*[MHEC-RAP] is only as good as the person behind the telephone and sometimes, I’ve been lucky enough, they’ve been fairly good, but there’s always the one that doesn’t realise the severity of the situation at the time. -2A*

*I suppose even though [MHEC-RAP have] done the assessment and they’ve got the documentation, can make you worry about somebody when they’ve been discharged and you don’t think that they’re right to go on their own. -1A*

The providers also felt MHEC-RAP was acceptable for their patients. Through MHEC-RAP, patients were getting the specialist help they needed. It was felt that patients could speak openly with MHEC-RAP and not feel stigmatised using the program. They were also able to maintain a level of privacy in their crisis by speaking with someone external because *“In a small country town … there’s no chance to be anonymous.” -1A*.

*They might not want to speak to me face-to-face, but they’re happy to speak to a complete stranger sometimes and discuss with them their own personal issues. -4A*

*We always give the patient the [freecall] number to take away as well in case they want to ring away from the hospital. -3A*

### Affordability

There were no direct costs borne by the providers to contact MHEC-RAP for help, and it was known to be free for the patients too, yet affordability of the program was discussed as providers identified other costs. MHEC-RAP was perceived to save the cost of unnecessary transportations of patients out of their community to another hospital thereby changing the previous culture of ‘schedule and transport’ to ‘assess and transport as needed’. The transportation of patients was significant for everyone because of variables of distance, time, and workforce implications.

*The only cost to us [is] transport, we have to call in people to transport and plus it’s the cost for us to transport by ambulance. I mean that’s $7900 just to drive them to [the nearest mental health inpatient unit], might be even a lot more than that. That’s where it comes to cost the system here. MHEC-RAP has reduced that. …so MHEC-RAP’s actually saved us money, a lot of money in transport fees. -4A*

*It does save money over a period of time because there are a number of times where we probably would have scheduled and sent somebody off, [with MHEC-RAP], they’ve assessed them and we could put that off for a period of time or assess whether it’s urgent that they go or not. -5B*

Although it was recognised that it may be necessary and appropriate to transport the patient for their safety and wellbeing, the financial cost for the patient and their family was also a consideration. Financial savings were believed to extend to the patient and their family when the patient was able to be cared for locally; specifically in respect to the expense for the patient’s return trip home or travel costs for the family to visit them in hospital.

*The most negativity is transport back. Because we ship them off there, but they can’t get back, so a lot of family, including the patient, say ‘oh I can’t go there’. …[They] do not wish to be voluntary patients. Family say ‘look they can’t go, we can’t get them back here’. -4A*

*Sometimes with that MHEC-RAP service they can deem that the person doesn’t need to be scheduled, they’re happy for them to go home … so it is a useful service in that way, not everybody obviously gets scheduled, but everybody gets, can get help through that service. -5B*

### Adequacy

Emergencies are unpredictable and the 24-hour structure of the program responded to deeply felt inadequacies of local specialist access, particularly afterhours and on weekends.

*[MHEC-RAP] is a fantastic service because we got quite a large mental health population and we didn’t have anything afterhours Monday to Friday … Having [MHEC-RAP] now 7 days-a-week, 24 hours-a-day is brilliant. -5B*

There were some aspects of MHEC-RAP that came under scrutiny; such as the video equipment used for clinical assessments and the layout of the local facilities. While the structure of the hospital ED is difficult to change, MHEC-RAP may respond to suggestions to modify the model of the video equipment to better meet local needs and concerns. For instance, large wall mounted systems could be exchanged with portable desktop devices bringing MHEC-RAP to the patient and possibly addressing local facility flaws.

*We’ve got a little lounge room at the back of ED [where the MHEC-RAP video is located], unfortunately, it’s not private, can’t shut the door … so it’s not as confidential as it could be and that’s a flaw in the design of our emergency department. -1A*

*Probably the biggest drawback is that [our MHEC-RAP system is] a fixed wall system … I think that it would be nice, as I said, occasionally if it were truly portable. -5B*

### Awareness

Only one provider had not heard of or used MHEC-RAP. Everyone else was aware and had some experience with the program. A few providers even identified it as part of their role to teach others locally about this resource, such as when, why, and how to use it.

*[The staff here] all know about it. …it’s more word of mouth. [MHEC-RAP hasn’t] changed much at all, they haven’t changed numbers, they do the same hours, the whole lot. -4A*

*I do a lot of orientation for the hospital, so I do make sure that they know about [MHEC-RAP] and the doctors, that’s part of our orientation with the doctors, you know even the locums that come in, that they know it’s there as well. -5B*

Reporting specific details of the program was fuzzy, such as where the service was located, but knowing this sort of detail is inconsequential. The providers who used the program were informed about its purpose and function. Some used MHEC-RAP infrequently while for others it became a routine part of their practice. The variation in their knowledge, understanding, and use of MHEC-RAP demonstrated local adaptation and flexibility of the model to meet need and complement existing systems.

*It depends, depends on the triage when they come through the door as to how we manage it. … Monday to Friday, during office hours, if we’ve got someone presenting to the emergency department with a mental health problem our first thing to do is to ring the community mental health team … it’s harder on the weekends and out of hours when we don’t have that, but we do have MHEC-RAP. -1A*

*If it’s a mental health presentation, we do MHEC-RAP. -6A*

*It helps when you do know about it, once you’ve used the MHEC-RAP a couple of times, you become, you know it does become your best friend afterhours especially … when you strike somebody who’s provided really sensible advice and so we use it a lot. -5B*

### Before and after

Before MHEC-RAP, providers felt alone, unsupported, and lacked confidence when dealing with emergency mental health presentations. They were mindful of long waiting times for assessments and concerned when patients were simply being medicated, involuntarily admitted to hospital, and unnecessarily transferred out of community which incurred costs of money, time, emotion, and human resources. Since MHEC-RAP, not all difficulties have been resolved, but access to specialist mental health care has changed the provision of such care locally. Many providers are using MHEC-RAP for every mental health presentation so that patients can get the right care with the assistance of a specialist. Some patients receive care plans that help direct local care and may reduce re-presentations. Providers also have access to much needed afterhour support. With MHEC-RAP, local providers have greater support and confidence to care for mental health patients.

*[MHEC-RAP will] actually guide us on what treatment what path to go down, whether it’s medication to give, that the patient’s right to go home, that they can be followed up by community mental health…or whether the patient needs scheduling and transferring. … I would think [MHEC-RAP] would have to reduce the level of re-presentation for someone who gets seen and treated [because] a care plan is started so that they go home. And it can make a difference, absolutely. -1A*

*MHEC-RAP’s made a big difference to the management of mental health problems to a point because you’re not then trying to do it by yourself … [MHEC-RAP] is providing a degree of reassurance. … Prior to [MHEC-RAP] you kind of felt like you were flying blind….despite the fact that we [see] a lot of [mental health]. … I think MHEC-RAP has changed the face of mental health in the bush. Reasonably in as much as it just gives us that afterhours support and so that helps significantly. I think it’s not the answer to everything and it doesn’t always work, and you can’t always put them in front of the camera and all that sort of thing, but it certainly helps significantly. -5B*

## Discussion and conclusions

Mental health emergencies are difficult to manage and required a different approach to patients presenting with physical ailments. Some local providers were not confident in their training and reluctant to provide care for such emergencies. Having access to MHEC-RAP who are able to conduct assessments, provide relevant and responsive information and advice on appropriate management and care of an emergency mental health presentation was invaluable. With MHEC-RAP, the local ED providers were no longer *‘flying blind’*.

MHEC-RAP was considered useful because there was a specialist on the other end of the telephone line who could help at any time of day. The program offered support and feedback to ED staff, while providing access to specialist assessment for their patients. MHEC-RAP not only enabled access to specialist emergency mental health care for these local providers, it changed their clinical practice and perspective. These providers reported a greater level of confidence to manage and care for mental health patients locally because of MHEC-RAP. They also felt that patient outcomes were improved, that fewer patients were being transferred for specialist mental health care, and that there were fewer representations of mental health patients in crisis. Initial studies support this, but further research is necessary to determine such outcomes [32,33].

Access is the degree of fit between the consumer and the service, and activity data from the evaluation showed that MHEC-RAP was well used by the ED providers, offering further evidence of access through use [33]. Understanding the experience of the program users through the lens of access, MHEC-RAP is able to identify modifications to ensure its services continue to be relevant and responsive to the needs of their consumers. For example, scheduling regular visits to communities across the region to promote the program and discuss local issues that impact on use of the service, as well as offering education sessions over the video link to remote local providers on a more frequent basis would support dialogue between MHEC-RAP and end users of the service. This would benefit access by raising awareness of the program and providing MHEC-RAP with feedback to further refine program delivery to ensure its acceptability and adequacy at the community level.

The experiences of these ED providers align with those reported by other providers in rural and remote communities [15,17-19,21,22,45-47]. Their access, use, and experience of MHEC-RAP suggest that the program could be implemented to address limitations in access to specialist care for emergency mental health presentations in other rural or remote communities.

The number of provider participants is small yet similar to other rural and remote research, however this may be considered to be a limitation as more participants would naturally provide more information. Still, this group of participants provided rich and useful information regarding their experience of emergency mental health care and their access to and use of MHEC-RAP with no new or differing information revealed over the course of the interviews. Personal participant details, such as age and time in place and occupation, were not directly collected. This may be considered a limitation in the data collection confining the degree of interpretation of the data and the detail of the participants reported. However, this and other analyses have yielded relevant interpretations that can inform program development and implementation.

For other areas considering the establishment of an emergency telepsychiatry service, assessing the fit between the consumer and service as a requirement for the development, evaluation, and ongoing management of the service would result in decisions about design and delivery that achieve improved access to care. While MHEC-RAP offers a practical and transferable model, assuring its sustainability also requires investigations into other potential impacts of the program, such as analysing patterns in patient transportations and cost-effectiveness. Additional research is also required to examine the experience of people with mental health conditions and their family and carers to determine the impact and ‘fit’ of MHEC-RAP with the broader community.

This study reports the change in practice through the experience of access to MHEC-RAP. Analysing provider experience through the lens of access confirms that the program is accessible and offers insights for those considering how to establish an emergency telepyschiatry service in other settings. MHEC-RAP has *“changed the face of mental health in the bush”* and it could transform the provision of emergency mental health care for providers and communities elsewhere.

## Abbreviations

ED: Emergency departments
GP: General practitioners
MHEC-RAP: Mental health emergency care-rural access program
WNSW/FWLHD: Western NSW and far west local health districts
